# *ε*‐Ga_2_O_3_: An Emerging Wide Bandgap Piezoelectric Semiconductor for Application in Radio Frequency Resonators

**DOI:** 10.1002/advs.202203927

**Published:** 2022-09-25

**Authors:** Zimin Chen, Xing Lu, Yujia Tu, Weiqu Chen, Zhipeng Zhang, Shengliang Cheng, Shujian Chen, Hongtai Luo, Zhiyuan He, Yanli Pei, Gang Wang

**Affiliations:** ^1^ School of Electronics and Information Technology Sun Yat‐Sen University Guangzhou 510006 China; ^2^ State Key Laboratory of Optoelectronic Materials and Technologies Sun Yat‐Sen University HEMC Guangzhou 510006 China; ^3^ Science and Technology on Reliability Physics and Application of Electronic Component Laboratory No.5 Electronics Research Institute of the Ministry of Industry and Information Technology Guangzhou 510610 China; ^4^ Foshan Institute of Sun Yat‐Sen University Foshan 528225 China

**Keywords:** gallium oxide, piezoelectricity, radio frequency resonator, surface acoustic waves

## Abstract

The explosion of mobile data from the internet of things (IoT) is leading to the emergence of 5G technology with dramatic frequency band expansion and efficient band allocations. Along with this, the demand for high‐performance filters for 5G radio frequency (RF) front‐ends keeps growing. The most popular 5G filters are constructed by piezoelectric resonators based on AlN semiconductor. However, AlN possesses a piezoelectric constant *d*_33_ lower than 5 pm V^−1^ and it becomes necessary to develop novel semiconductors with larger piezoelectric constant. In this work, it is shown that strong piezoelectricity exists in *ε*‐Ga_2_O_3_. High‐quality phase‐pure *ε*‐Ga_2_O_3_ thin films with a relatively low residual stress are prepared. A switching spectroscopy piezoelectric force microscope (SS‐PFM) measurement is carried out and the piezoelectric constant *d*_33_ of *ε*‐Ga_2_O_3_ is determined to be ≈10.8–11.2 pm V^−1^, which is twice as large as that of AlN. For the first time, surface acoustic wave (SAW) resonators are demonstrated on the *ε*‐Ga_2_O_3_ thin films and different vibration modes resonating in the GHz range are observed. The results suggest that *ε*‐Ga_2_O_3_ is a great material candidate for application in piezoelectric devices, thanks to its wide bandgap, strong piezoelectric property, small acoustic impedance, and low residual stress.

## Graphical abstract

The piezoelectricity in *ε*‐Ga_2_O_3_ is demonstrated. The piezoelectric coefficient *d*_33_ of *ε*‐Ga_2_O_3_ (≈10.8–11.2 pm V^–1^) is twice larger than that of AlN. For the first time, *ε*‐Ga_2_O_3_‐based surface acoustic wave (SAW) radio‐frequency resonators are demonstrated. The wide bandgap and high piezoelectricity make *ε*‐Ga_2_O_3_ a strong competitor to traditional piezoelectric semiconductors in the communication industry.

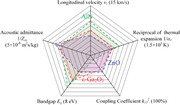

## 1 Introduction

During the past decade, the rapid evolution of wireless communication technologies has led to the urgent requirement for wide bandwidth and high‐frequency data transmissions. The emerging 5G networks have been greatly improved with a high‐level utilization of spectrum resources and communication protocols in the GHz range. Today, more than 30 bands are being used, and this number keeps growing, resulting in a consumption of over 10 billion RF filters per year. For this reason, there is an urgent demand for the development of high‐performance piezoelectric resonators, which are the basic component of RF filters in communication systems. Proven with commercial success, the SAW resonators based on piezoelectric crystals LiNbO_3_ or LiTaO_3_ are the mainstream for low‐frequency bands, while the high‐frequency applications are dominated by the bulk acoustic wave (BAW) resonators made of AlN semiconductor thin films.^[^ ^1^, ^2^ ^]^

Resonators made of piezoelectric semiconductor thin films are of special interest since their fabrication process is compatible to the modern semiconductor industry. There are two criteria for the piezoelectric semiconductors to be utilized in acoustic resonators: a wide bandgap *E*_g_ and a high electromechanical coupling coefficient *k* ^2^. The former gives a high material resistivity and therefore benefits for the device quality factor (Q) and power handling capability, while the latter contributes to a wide bandwidth.^[^ ^3^ ^]^ The AlN thin film was first proposed for fabricating acoustic resonator in 1982 and it has become the mainstream material for commercial 5G filters.^[^ ^4^ ^]^ AlN possesses a wide enough bandgap (*E*_g_ = 6.2 eV) to guarantee high intrinsic resistivity, while it suffers from a low piezoelectric constant of only ≈1–5 pm V^−1^,^[^ ^5^, ^6^, ^7^, ^8^, ^9^, ^10^, ^11^ ^]^ which means a compromised electromechanical coupling coefficient *k*_33_^2^. So far, however, no better piezoelectric semiconductors have been found by the industry to replace AlN.

Incorporation of scandium into AlN films is able to enhance the piezoelectric constant and a *d*_33_ value larger than 20 pm V^−1^ has been achieved in the AlScN thin films with a high Sc composition of over 40%.^[^ ^12^ ^]^ A high Sc composition is necessary in order to increase the piezoelectric coefficient of AlScN. However, limited by the solid solubility of Sc in AlN, it is very difficult to deposit AlScN thin film with high Sc composition and high composition uniformity at the same time.^[^ ^12^, ^13^ ^]^ Therefore, the composition segregation in the AlScN thin film may cause a hazard to manufacturing yield. ZnO thin film is another candidate for RF resonators due to its high piezoelectric constant *d*_33_ exceeding 10 pm V^−1^.^[^ ^8^ ^]^ Unfortunately, the bandgap of ZnO (*E_g_* = 3.3 eV) is not wide enough to guarantee a high resistivity. ZnO thin films, in most instances, show an n‐type conductivity originating from point defects like oxygen vacancies or hydrogen impurity.^[^ ^14^, ^15^ ^]^

Recently, Ga_2_O_3_ semiconductor has attracted lots of research attention for application in power devices due to its wide bandgap (*E_g_* = 4.9 eV) and high breakdown electric field (8 MV cm^−1^).^[^ ^16^, ^17^ ^]^ There are five different phases of Ga_2_O_3_ including *α*‐, *β*‐, *γ*‐, *δ*‐, and *ε*‐Ga_2_O_3_. *β*‐Ga_2_O_3_, which belongs to monoclinic structure, is the most stable phase of Ga_2_O_3_ and its bulk crystal could be grown by melt method.^[^ ^18^ ^]^ *ε*‐Ga_2_O_3_, also known as *κ*‐Ga_2_O_3_, is the second most stable phase of Ga_2_O_3_, which could only be grown by heteroepitaxy.^[^ ^19^, ^20^ ^]^ Predicted by the theoretical studies, the asymmetric lattice structure (space group *Pna2_1_*) brings strong spontaneous polarization in *ε*‐Ga_2_O_3_ with a spontaneous polarization coefficient *P*_sp_ of ≈23–79 µC cm^−2^ along the *c*‐axis, which is about one order higher than that of AlN.^[^ ^21^, ^22^, ^23^, ^24^ ^]^ In addition, the piezoelectric polarization is also predicted in *ε*‐Ga_2_O_3_.^[^ ^22^, ^23^, ^24^ ^]^ Similar to the established III‐nitride semiconductors, the strong polarization effects in *ε*‐Ga_2_O_3_ make it promising for applications both in electronic and piezoelectric devices.^[^ ^25^, ^26^ ^]^ However, due to the difficulties in the growth of high‐quality and phase‐pure *ε*‐Ga_2_O_3_ thin films, there is no experimental proof or application of the polarization effects in *ε*‐Ga_2_O_3_.

In this work, high‐quality *ε*‐Ga_2_O_3_ thin films were grown on Sapphire and Si substrates by metal‐organic chemical vapor deposition (MOCVD). The material properties of *ε*‐Ga_2_O_3_ were investigated thoroughly and a large piezoelectric coefficient of *d*_33_= 10.8∼11.2 pm V^−1^ is detected. The *ε*‐Ga_2_O_3_‐based SAW piezoelectric resonators were demonstrated for the first time and the devices resonate at the Rayleigh mode and Sezawa modes in the range of ≈1–3 GHz. Based on the results, we propose *ε*‐Ga_2_O_3_ as a favorable piezoelectric semiconductor for 5G RF filters.

## 2 Results and Discussion

**Figure 1:**
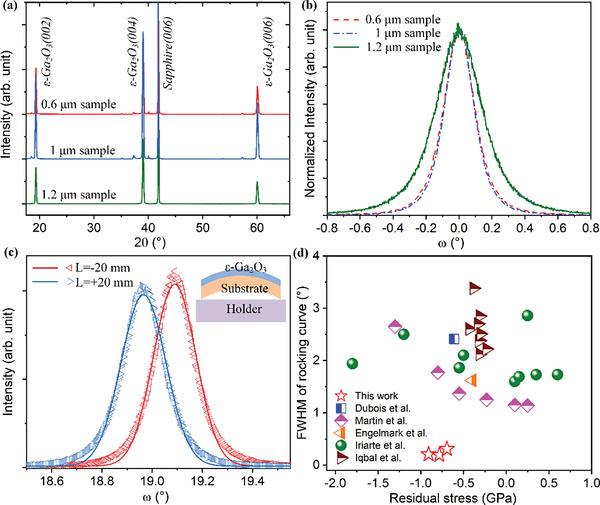
a) 2*θ*‐scan of the 0.6, 1, and 1.2 µm *ε*‐Ga_2_O_3_ samples. The diffraction spectra are shifted along the *y*‐axis to distinguish clearly. b) Rocking curve of *ε*‐Ga_2_O_3_ (004) plane. The diffraction intensities are normalized and centered at *ω* = 0. c) Rocking curve of the (004) plane for the 1‐µm‐thick sample at the left (L = +20 mm) and right (L = −20 mm) sides of the wafer. Solid lines are the Gaussian fits of the experimental data. The inset shows the convex wafer bowing. d) Comparison of the crystalline quality and residual stress between the *ε*‐Ga_2_O_3_ and AlN thin films. The rocking curve measurement of AlN is carried out on (002) plane.^[^ ^6^, ^7^, ^30^, ^31^, ^32^ ^]^

The structural properties of the *ε*‐Ga_2_O_3_ thin films grown on Sapphire substrates were investigated by high‐resolution X‐ray diffraction (HRXRD) and Transmission electron microscope (Figure S1, Supporting Information). As shown in **Figure** **1**a, the diffraction peak at ≈41.85° corresponds to Sapphire(006) plane, while diffraction peaks at 19.30°, 39.03°, and 60.05° corresponds to the (002), (004), and (006) plane of *ε*‐Ga_2_O_3_, respectively.^[^ ^27^, ^28^ ^]^ All the samples presented an epitaxial relationship of *ε*‐Ga_2_O_3_(001)//Sapphire(0001). Typical diffraction peaks originating from *β*‐Ga_2_O_3_ were not observed in these samples,^[^ ^29^ ^]^ indicating that phase‐pure *ε*‐Ga_2_O_3_ with uniform *c*‐axis orientation have been obtained in this study. The crystal quality was examined by rocking curve measurement of *ε*‐Ga_2_O_3_(004). As shown in Figure 1b, the full width of half maximum (FWHM) of the *ω*‐scan is 0.21°, 0.19°, and 0.31° for the samples with thickness of 0.6, 1, and 1.2 µm, respectively.

For wafer bowing measurement, the holder moved horizontally by *L* = +/−20 mm from the center to perform the *ω*‐scan of *ε*‐Ga_2_O_3_(004) at the left/right side of the wafer, deriving a diffraction peak at *ω*_L_/*ω*_R_ (Figure S2, Supporting Information). For the 1‐µm‐thick sample, the *ω*‐scans performed at both sides are shown in Figure 1c. The difference of diffraction peak Δ*ω* = *ω*_R_ − *ω*_L_ = −0.124° corresponds to a convex wafer curvature *κ* = 1/*R* = −0.054 m^−1^ (inset of Figure 1). The stress could be calculated according to Stoney's Equation:

(1) $$\sigma \;=\frac{E_{s}}{6\left(1-\nu \right)}\;\frac{t_{s}^{2}}{t_{f}}\left(\frac{1}{R}-\frac{1}{R_{0}}\right)$$

with *E*_s_ = 380 GPa and *ν =* 0.28 the Young's modulus and Poisson's ratio of Sapphire, *t*_s_ and *t*_f_ the thickness of substrate and thin film. 1/*R*_0_ = 0 and 1/*R* ≈ Δ*ω*/2|*L*| is the curvature of the substrate before and after the growth of *ε*‐Ga_2_O_3_. The residual stress for the samples with thickness of 0.6, 1, and 1.2 µm is −0.91, −0.78, and −0.69 GPa, respectively (Figure S3, Supporting Information).

Residual stress is a key factor for the mass production of BAW filters. As the mainstream semiconductor material for BAW filters, AlN thin films are usually deposit by Magnetron Sputtering at a low temperature to minimize the residual stress to a sub‐GPa level.^[^ ^7^, ^30^, ^31^, ^32^ ^]^ However, the low deposition temperature leads to a poor crystal quality of AlN and a low piezoelectric coefficient *d*_33_ of only ≈1–5 pm V^−1^.^[^ ^5^, ^6^, ^7^, ^8^, ^9^ ^]^ On the other hand, the *ε*‐Ga_2_O_3_ thin films in this study are grown at a medium temperature of about 600 °C. The crystal quality, typically reflected by the FWHM of rocking curve, is much better than the sputtered AlN thin films. A comparison of the crystal quality and residual stress between the *ε*‐Ga_2_O_3_ and AlN thin films is made in Figure 1d. Data of AlN are collected from literatures, where AlN thin films were deposit on Si substrates by Magnetron Sputtering. The FWHM of AlN(002) rocking curve is rarely lower than 1°, while it is only ≈0.19°–0.31° for the *ε*‐Ga_2_O_3_(004) in this study. Moreover, the *ε*‐Ga_2_O_3_ thin films, though grown at a higher temperature than the sputtered AlN, present a comparably low residual stress of sub‐GPa level.

**Figure 2:**
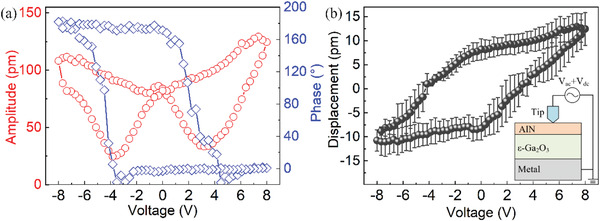
a) *V*_dc_‐dependence of the phase and amplitude of PFM measurement. b) *V*_dc_‐dependence of the piezoelectric displacement of the AlN/*ε*‐Ga_2_O_3_ sample.

The piezoelectric properties of *ε*‐Ga_2_O_3_ thin film are investigated by switching spectroscopy Piezoelectric Force Microscope (SS‐PFM) measurements in **Figure** **2**. It should be noticed that the sample used for PFM measurement were grown on Silicon substrate instead of Sapphire due to the difficulty of substrate lift‐off. For the *ε*‐Ga_2_O_3_ grown on Si(111), a 50‐nm‐thick AlN insertion layer was deposit at first to avoid surface oxidation of the Si substrate.^[^ ^33^ ^]^ During the measurement, the metal substrate was grounded and the voltage was applied on the surface through the PFM tip, as illustrated in the inset of Figure 2b. The DC voltage *V*_dc_ applied on the sample scanned through −8 V → 0 V → +8 V → 0 V → −8 V. An AC signal of *V*_ac_ = 0.8 V was also superimposed to induce oscillation of the sample along the *c*‐axis (Figure S4, Supporting Information). The measurements were carried out in four different positions on the sample and each position was measured by two times (Figure S5, Supporting Information). All the results shown in Figure 2 are averaged values.

It could be observed in Figure 2a that the phase *φ* and amplitude *A* of the sample oscillation are *V*_dc_‐dependent and a butterfly‐like characteristic of *A*(*V*_dc_) is obtained. The displacement of the sample caused by piezoelectricity is defined as *d*_pz_ *(V*_dc_) = *A*cos*φ*/G. The gain of the equipment G is 10, which is determined by calibrating the displacement on a standard quartz sample. The hysteresis loop shown in Figure 2b may originate from either ferroelectricity^[^ ^34^, ^35^ ^]^ or Maxwell force effect.^[^ ^36^ ^]^ It should be noticed that ferroelectricity was also observed in some materials used for commercial RF resonators such as LiNbO_3_.^[^ ^37^ ^]^ The piezoelectric measurement of a pure AlN layer shows very weak hysteresis characteristics so that the *ε*‐Ga_2_O_3_ layer is the main contributor of the piezoelectricity characteristics detected in Figure 2.^[^ ^9^ ^]^ The displacement at *V*_dc_ = 0 V is *d*_pz_(0 V) = (8.19+|−8.23|)/2 pm = 8.21 pm in Figure 2b. The piezoelectric strain constant *d*_33_ could be extracted by *d*_33_ = *d*_pz_(0 V)/*V*_ac_ = 10.26 pm V^−1^.^[^ ^34^, ^38^ ^]^

Note that the piezoelectric measurement was carried out on the AlN/*ε*‐Ga_2_O_3_ heterostructure, i.e., a combination of 50‐nm thick AlN layer and 360‐nm thick *ε*‐Ga_2_O_3_ layer. However, the above analysis neglects the effect of the AlN layer on top. The AlN layer contributes to the measurement through *V*_ac_ = *V*_ac,AlN_ + *V*_ac,Ga2O3_ = 0.8 V and *d*_pz_(0 V) = *d*_pz,AlN_ +*d*_pz,Ga2O3_ = 8.21 pm. Considering the dielectric constants of *ε*_AlN_ = 10 and *ε*_Ga2O3_  = 13.2, the AC voltage applied on the two layers should obey *V*_ac,AlN_:*V*_ac,Ga2O3_ = (*ε*_AlN_ *t*_AlN_): (*ε*_Ga2O3_ *t*_Ga2O3_), with *t* the thickness of different layers. The *d*_33_ of AlN depends greatly on the crystalline quality and the uniformity of AlN(001) orientation. The typical value is ≈1–5 pm V^−1^. The *d*_33_ value of *ε*‐Ga_2_O_3_ is then calculated to be ≈10.8–11.2 pm V^−1^, which is about twice as large as AlN.

**Figure 3:**
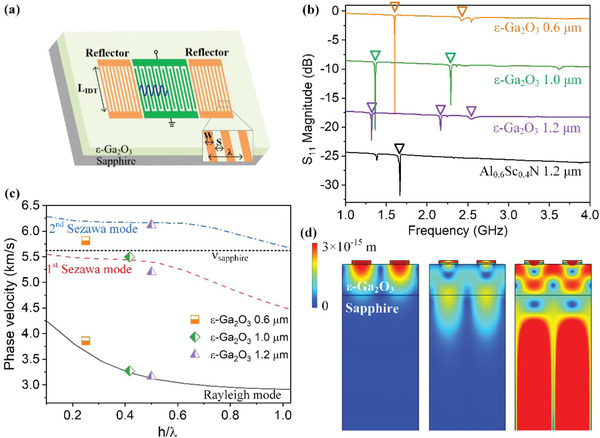
a) Schematic diagram of the *ε*‐Ga_2_O_3_ SAW resonator. b) *S*_11_ parameters of the SAW resonator fabricated on different samples. Different curves are down‐shifted by a magnitude of 0, 8, 16, and 24 dB to distinguish clearly. The identified Rayleigh and Sezawa modes are marked by empty triangles. c) Dispersion characteristics of the acoustic wave velocity for *ε*‐Ga_2_O_3_/Sapphire structure calculated by FEM. The wavelength is fixed at *λ* = 2.4 µm. The experimental values are also shown in the figure. The dot line represents the slow shear wave velocity of Sapphire. d) FEM calculation of the distribution of vibration amplitude at resonance frequencies. From left to right: 1.30 GHz (Rayleigh mode), 2.25 GHz (first‐order Sezawa mode), 2.57 GHz (second‐order Sezawa mode).

To further verify the piezoelectricity in *ε*‐Ga_2_O_3_, the single‐port SAW resonators were fabricated using phase‐pure *ε*‐Ga_2_O_3_ thin films grown on sapphire, as schematically shown in **Figure** **3**a. The acoustic waves were excited by the interdigital transducers (IDTs) with wavelength *λ* = 2.4 µm. The measured S_11_ parameters of the SAW resonators are shown in Figure 3. Resonance of acoustic wave is observed for all the samples in the range of ≈1–3 GHz, demonstrating the existence of piezoelectricity in *ε*‐Ga_2_O_3_. The phase velocity of excited acoustic wave is determined by the resonance frequency and wavelength with *v*_phase_ = *λf*. The fundamental Rayleigh mode located at 1.61, 1.36, and 1.32 GHz for the three samples with different thickness, corresponding to the phase velocity of 3.86, 3.26, and 3.17 km s^−1^, respectively. For piezoelectric film with a higher thickness‐to‐wavelength ratio (typically *h*/*λ* > 0.3),^[^ ^39^ ^]^ high order vibration modes could be observed besides Rayleigh mode. Such phenomenon was also observed in our study with *ε*‐Ga_2_O_3_ film thickness of 1 and 1.2 µm, as shown in Figure 3b. The first‐order Sezawa mode vibration is clearly observed at 2.29 GHz on the 1‐µm‐thick sample. For the 1.2‐µm‐thick sample, the first‐order and second‐order Sezawa modes resonated at 2.19 and 2.54 GHz, respectively. As a comparison, SAW resonator was also fabricated on the AlScN thin film sputtered on Sapphire substrate. The Rayleigh mode vibration resonates at 1.67 GHz for device period of *λ* = 2.4 µm, corresponding to a phase velocity of 4.01 kms^−1^.

The velocity diversion of acoustic wave for the *ε*‐Ga_2_O_3_‐on‐Sapphire was further investigated by using Finite Element Method (FEM). It should be noted that many basic parameters of *ε*‐Ga_2_O_3_ have not yet been determined due to the difficulties in the growth of phase‐pure sample. In this work, the elastic stiff tensors of *ε*‐Ga_2_O_3_ were set according to first principle calculations as listed in **Table** **1**,^[^ ^19^, ^23^ ^]^ while the mass density *ρ* was treated as a variable. The density of *ε*‐Ga_2_O_3_ was determined to be 4.8 gcm^−3^ through fitting the resonance frequencies calculated by FEM with the experimental data (Figures S7 and S8, Supporting Information). The dependence of phase velocity on h/*λ* extracted by FEM is shown in Figure 3c for both Rayleigh mode and Sezawa modes. Velocities at resonance frequencies for the three samples are also displayed in the figure. The velocity of the slow shear acoustic wave for sapphire is ≈5.65 kms^−1^ [indicated by dot line in Figure 3c], thus vibrations with phase velocities higher than 5.65 km s^−1^ could couple to substrate vibration and become a leaky mode. For the h/*λ* range investigated in Figure 3c, the second‐order Sezawa mode is a leaky mode. This could explain why the second‐order Sezawa resonance is extremely weak for the 1.2‐µm‐thick sample, and even absent for the samples with thickness of 0.6 and 1 µm. It should also be noticed that the phase velocity of AlScN is much higher than that of *ε*‐Ga_2_O_3_. For this reason, the Sezawa modes in the AlScN SAW device are leaky modes and their resonations are invisible in Figure 3b.

**Table 1:** Parameters of AlN, *ε*‐Ga_2_O_3_, and ZnO

	AlN	*ε*‐Ga_2_O_3_	ZnO
Bandgap *E_g_* (eV)	6.2	4.9^a)^	3.3
Density *ρ* (g cm^−3^)	3.26	4.8^b)^	5.61
*d*_33_ (pm V^−1^)	5^[^ ^8^ ^]^	≈10.8–11.2^c)^	12.3^[^ ^8^ ^]^
Permittivity *ε*	10^[^ ^16^ ^]^	13.2^[^ ^22^ ^]^	8.8^[^ ^8^ ^]^
*C*_11_ (GPa) ^[^ ^14^, ^19^, ^40^ ^]^	396	312	207
*C*_13_ (GPa) ^[^ ^14^, ^19^, ^40^ ^]^	108	126.2	106.1
*C*_33_ (GPa) ^[^ ^14^, ^19^, ^40^ ^]^	373	279.8	209.5
Young's modulus *E* (GPa)^d)^	314.4	188.8	125
Poisson's ratio *v* ^d)^	0.2475	0.3309	0.3534
Acoustic impedance *Z_ac_* (10^7^ kg m^−2^ s^−1^)^e)^	3.2	3.0	2.6
Longitudinal wave velocity *v_l_* (km s^−1^)^d)^	10.2	7.6	6.0
Shear wave velocity vs (km s^−1^)^d)^	5.9	3.8	2.9
Volume thermal expansion *α_V_* at 300 K (10^−5^ K^−1^)	1.1^[^ ^41^ ^]^	1.4^[^ ^42^ ^]^	1.5^[^ ^43^ ^]^

^a)^ Derived from optical transmittance, see Figure S9 (Supporting Information);

^b)^ Derived by fitting the FEM calculation results with the experiments, see Figure S8 (Supporting Information);

^c)^ Derived from SS‐PFM measurement;

^d)^ Mechanical properties were calculated by Voigt‐Reuss‐Hill model, see Supporting Information;

^e)^ *Z*_ac_ = (*ρE*)^1/2^.

The distributions of vibration amplitude for the 1.2‐µm‐thick sample resonating at different modes are shown in Figure 3d. The vibration of Rayleigh mode is mainly confined at the surface of *ε*‐Ga_2_O_3_ thin film, while the vibration of the first‐order Sezawa mode distributes down to the *ε*‐Ga_2_O_3_/Sapphire interface. The vibration changes pronouncedly for the second‐order Sezawa mode, where most of the vibration energy penetrates into the substrate. This result is in consistence with the above conclusion that the second‐order Sezawa mode is a leaky mode due to its high propagating velocity.

**Figure 4:**
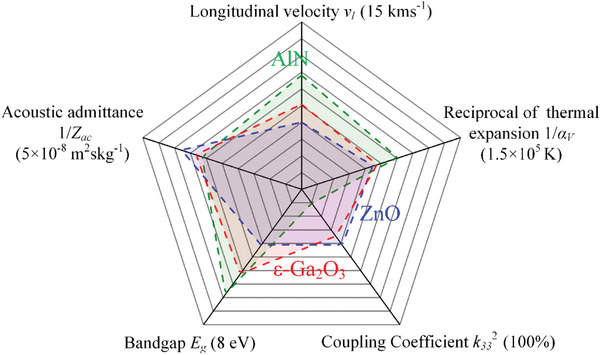
Comparison of piezoelectric semiconductors AlN, *ε*‐Ga_2_O_3_, and ZnO.

In **Figure** **4**, a comprehensive comparison is made between *ε*‐Ga_2_O_3_ and the other two mainstream wide bandgap piezoelectric semiconductors AlN and ZnO. Details of the parameters are also listed in Table 1. For the piezoelectric semiconductor thin films grown along (001) direction, piezoelectric constants *d*_33_ and *d*_31_ are of special importance. For BAW resonators, *d*_33_ plays a critical role since the electromechanical coupling coefficient is defined by *k*_33_^2^ *= d*_33_^2^/*s*_33_ *ε*_33_, with *s*_ij_ the elastic compliance coefficient and *ε*_ij_ the dielectric constant. While in SAW resonators, it is *d*_31_ that determines the electromechanical coupling coefficient by *k*_31_^2^ *= d*_31_^2^ */s*_33_ *ε*_11_. The extracted *d*_33_ of *ε*‐Ga_2_O_3_ is two times larger than that of AlN, which translates to a four times higher electromechanical coupling coefficient *k*_33_^2^ of *ε*‐Ga_2_O_3_ compared to AlN. This characteristic demonstrates that *ε*‐Ga_2_O_3_ has a great potential for application in piezoelectric devices, especially BAW resonators.

The material bandgap and piezoelectricity, to some extent, are a pair of competing parameters, so there are barely any semiconductors exhibiting a wide bandgap and a high piezoelectricity at the same time. Generally, a wide bandgap originates from the strong chemical bonds between atoms, which may lead to a larger elastic constant and a high hardness. For example, the comparisons of bandgaps and Young's modulus among AlN, *ε*‐Ga_2_O_3_ and ZnO are *E*_g,AlN_ > $E_{g,{\mathrm{Ga}}_{2}O_{3}}>E_{g,\mathrm{ZnO}}$ and $E_{\mathrm{AlN}}>E_{{\mathrm{Ga}}_{2}O_{3}}$ > *E*_ZnO_ (see Table 1), respectively. However, the hard materials tend to show a low piezoelectric constant since they deform more difficultly under external force. AlN has a wide bandgap and therefore a sufficiently high intrinsic resistivity, but it faces the problem of low electromechanical coupling coefficient originating from its low piezoelectric constant *d*_33_ of only ≈1–5 pm V^−1^. ZnO possesses a high piezoelectric constant *d*_33_ of ≈12.4 pm V^−1^, while its bandgap is not wide enough to guarantee a high resistivity. Even though the bandgap of *ε*‐Ga_2_O_3_ is lower than that of AlN, our previous work has shown that *ε*‐Ga_2_O_3_ thin films grown by MOCVD possess a resistivity of ≈10^4^ Ωcm, which is high enough for RF resonator application.^[^ ^44^ ^]^ To date, for the fabrication of RF resonators, AlN thin film is usually grown by Magnetron Sputtering at low temperature,^[^ ^30^, ^31^, ^32^ ^]^ which is much easier than the MOCVD growth of *ε*‐Ga_2_O_3_ investigated in this work. However, *ε*‐Ga_2_O_3_ has demonstrated such a unique property that it possesses a good balance between the bandgap (resistivity) and piezoelectricity and is a better material choice for application in RF resonators.

The thermal expansion coefficient *α_V_* and the acoustic impedance *Z*_ac_ = (*ρE*)^1/2^ are another two important parameters for BAW resonators. The thermal expansion coefficient *α_V_* mainly determines the thermal stability of resonators. The acoustic impedance *Z*_ac_ greatly affects the quality factor Q and the effective electromechanical coupling coefficient *k*_eff_^2^ of the devices. To better confine the vibration energy within the piezoelectric layer of a BAW resonator, *Z*_ac_ should be as low as possible compared to the metal electrodes.^[^ ^2^ ^]^ The acoustic impedance of *ε*‐Ga_2_O_3_ is 3 × 10^7^ kg m^−2^ s^−1^, slightly lower than that of AlN, which means that the metallization strategy in commercial AlN resonators is also suitable for *ε*‐Ga_2_O_3_ resonator.

## 3 Conclusion

In conclusion, high‐quality *ε*‐Ga_2_O_3_ thin films grown by MOCVD heteroepitaxy have been demonstrated and it is proposed as an emerging piezoelectric semiconductor for the application of RF resonator. The *ε*‐Ga_2_O_3_ thin films presented a sub‐GPa residual stress, which is comparable to the sputtered AlN thin films. The SS‐PFM measurement was carried out on a transferred *ε*‐Ga_2_O_3_/AlN heterostructure (360 nm/50 nm) and the piezoelectric strain constant *d*_33_ of *ε*‐Ga_2_O_3_ is extracted to be ≈10.8–11.2 pm V^−1^, which is twice as large as that of AlN. The piezoelectricity in *ε*‐Ga_2_O_3_ is further demonstrated by the fabrication and characterization of SAW resonator. Different vibration modes resonating at ≈1–3 GHz were observed in the *ε*‐Ga_2_O_3_ resonators. Combining the FEM calculation and experiments, the mass density and SAW velocity dispersion of *ε*‐Ga_2_O_3_ were analyzed, and its acoustic impedance is calculated to be 3 × 10^7^ kg m^−2^ s^−1^. The *ε*‐Ga_2_O_3_ thin films investigated in this work present a low residual stress, a small acoustic impedance, a wide bandgap and a high piezoelectric constant, demonstrating a great potential for application in microwave acoustic resonators. These properties also make *ε*‐Ga_2_O_3_ a potentially strong competitor to traditional piezoelectric semiconductors in the communication industry.

## 4 Experimental Section

### Growth of *ε*‐Ga_2_O_3_ Thin Films

The *ε*‐Ga_2_O_3_ thin films were grown on 2‐inch c‐plane Sapphire or Si(111) substrates in a home‐made MOCVD system. Due to the large lattice mismatch between *ε*‐Ga_2_O_3_ and the substrate, a two‐step growth method was used: a nucleation layer with thickness of ≈30–100 nm was first deposit at ≈530–560 °C, followed by an epilayer grown at ≈600–640 °C with a thickness of ≈330–1100 nm. Deionized water (H_2_O), Triethylgallium (TEGa), and Argon were used as the precursor of O, Ga, and carrier gas, respectively. The growth was carried in oxygen‐rich condition with VI/III ratio of ≈100. Four samples were prepared with total thickness of 360, 600, 1000, and 1200 nm. The 360‐nm‐thick sample was grown on Si(111) substrate, while the others were grown on sapphire substrate. For the sample grown on Si(111) substrate, a 50‐nm‐thick AlN insertion layer was deposit at first to avoid surface oxidation of the Si substrate during the growth of Ga_2_O_3_. More details of the *ε*‐Ga_2_O_3_ sample growth on Sapphire and Si(111) substrates could be found in our previous work.^[^ ^28^, ^33^ ^]^

### HRXRD Measurements

HRXRD was used for rocking curve and wafer bowing measurements for the samples grown on sapphire substrates. Experiments were carried out in a Bruker D8 Discover system equipped with a four‐bounce Ge(220) monochromator. The X‐ray wavelength was *λ* = 0.154160 nm and the slit width was 0.5 mm for diffraction beam detection. The sample holder allowed sample rotation with *ω*‐, *φ*‐, *χ*‐angle, and horizontal movement in the x‐y plane. For wafer bowing and residual stress measurement, the sample holder moved horizontally along x‐direction by L = +/−20 mm to perform *ω*‐scan at the edge of the wafer (Figure S1, Supporting Information).

### SS‐PFM Measurements

The PFM measurements were performed on a commercial atomic force microscope (Cypher, Asylum Research) with Pt‐coated silicon tips (EFM Arrow, Nanoworld). The SS‐PFM measurement required a metal electrode at bottom of the thin film. The *ε*‐Ga_2_O_3_ samples grown on Sapphire substrates were not suitable because it was too difficult to remove the sapphire substrate and expose the backside of the *ε*‐Ga_2_O_3_ thin film. Instead, the *ε*‐Ga_2_O_3_ sample grown on Si(111) substrate was used. A 50‐nm‐thick AlN insertion layer was deposit at first to avoid surface oxidation of the Si substrate during the growth of Ga_2_O_3_.^[^ ^33^ ^]^ The sample was flipped upside down and bonded onto a metal substrate. Then, the original Si (111) substrate was removed by mechanical grinding and inductively coupled plasma (ICP) etching to expose the *ε*‐Ga_2_O_3_/AlN heterostructure for the SS‐PFM measurement [see the inset of Figure 2b].

### SAW Device Fabrication

The SAW resonators were fabricated using *ε*‐Ga_2_O_3_ samples grown on Sapphire substrates. Commercial Al_0.6_Sc_0.4_N thin film sputtered on Sapphire substrate with a thickness of 1.2 µm was also used as a comparison. The IDT electrodes were formed by electron beam lithography, Ni/Au (60/90 nm) metal evaporation, and lift‐off process. The IDTs were composed of 80 pairs of metal strips aligned and connected to the busbars periodically, while the grating reflectors were composed of 60 pairs of shorted metal strips. The finger width *W*, finger spacing *S*, and finger length *L*_IDT_ of the IDT were 0.6, 0.6, and 192 µm, respectively. The wavelength of the SAW resonators *λ* is 2.4 µm. The RF response of the SAW resonators was measured using a network analyzer in the frequency range of ≈1–4 GHz. The phase velocity of acoustic wave was analyzed by FEM calculation with COMSOL software. Details of the FEM model could be found in Figure S7 (Supporting Information).

## Conflict of Interest

The authors declare no conflict of interest.

## Supporting information

Supporting information

## Data Availability

The data that support the findings of this study are available from the corresponding author upon reasonable request.
