# Supplementary material for: ε‐Ga2O3: An Emerging Wide Bandgap Piezoelectric Semiconductor for Application in Radio Frequency Resonators
Source: Adv Sci (Weinh). 2022 Sep 25;9(32):2203927. doi: 10.1002/advs.202203927 (PMC9661831; doi:10.1002/advs.202203927)
Supplement: Supplementary file 1 — Supporting information [file ADVS-9-2203927-s001.pdf]

ADVANCED SCIENCE
Open Access

Supporting Information

for *Adv. Sci.*, DOI 10.1002/advs.202203927

ϵ-Ga_2O_3: An Emerging Wide Bandgap Piezoelectric Semiconductor for Application in Radio Frequency Resonators

Zimin Chen, Xing Lu, Yujia Tu, Weiqu Chen, Zhipeng Zhang, Shengliang Cheng, Shujian Chen, Hongtai Luo, Zhiyuan He, Yanli Pei and Gang Wang**

ε-Ga_2O_3: an Emerging Wide Bandgap Piezoelectric Semiconductor for Application in Radio Frequency Resonators

Zimin Chen Xing Lu, Yujia Tu, Weiqu Chen, Zhipeng Zhang, Shengliang Cheng, Shujian Chen, Hongtai Luo, Zhiyuan He, Yanli Pei and Gang Wang

Z. Chen, X. Lu, Y. Tu, W. Chen, Z. Zhang, S. Cheng, S. Chen, H. Luo, Y. Pei, G. Wang
School of Electronics and Information Technology, Sun Yat-Sen University, Guangzhou 510006, China.
E-mail: lux86@mail.sysu.edu.cn; stswangg@mail.sysu.edu.cn

Z. Chen, X. Lu, Y. Pei, G. Wang
State Key Laboratory of Optoelectronic Materials and Technologies, Sun Yat-Sen University, HEMC, Guangzhou 510006, China.

Z. He
Science and Technology on Reliability Physics and Application of Electronic Component Laboratory, No.5 Electronics Research Institute of the Ministry of Industry and Information Technology, Guangzhou 510610, China

G. Wang
Foshan Institute of Sun Yat-Sen University, Foshan 528225, China

1. TEM measurement

The 1.2-μm-thick ε-Ga_2O_3 sample was characterized by TEM in Figure S1. The film is pure ε-phase confirmed by the selected area electron diffraction (SAED) in Figure S1(b), where the diffractions of (002) and (030) planes are indicated. High resolution TEM images in Figure (d) and (e) present clear periodic arrangement of atoms, indicating the high crystal quality of the ε-Ga_2O_3 film.

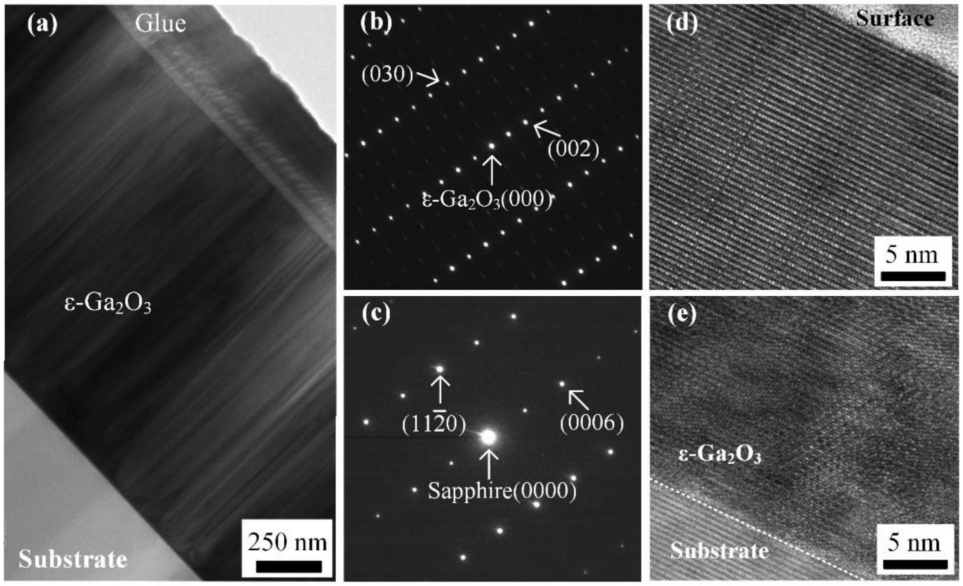

Figure S1. TEM measurement of the 1.2-μm-thick ε-Ga_2O_3 sample. (a) The whole film. SAED of (b) the ε-Ga_2O_3 film and (c) the substrate. Magnified image of the ε-Ga_2O_3 film near the (d) surface and (e) film/substrate interface.

2. Measurement of wafer bowing and residual stress by XRD

For wafer bowing measurement, the ω-scan of ε-Ga_2O_3(004) plane was first carried out in the center of the wafer, deriving a diffraction peak at ω_c. Then the holder moved by L=+/-20 mm along the x-direction to perform ω-scan at the left/right side of the wafer, deriving the diffraction peak at ω_L/ω_R. The diffraction geometry in Fig. S1(b) shows the measurement at the left side of the wafer. The curvature of the wafer is hence defined by $\kappa \approx \frac{\omega_R - \omega_L}{2|L|} = \frac{\Delta\omega}{2|L|}$. The wafer bowing height H could be calculated by H≈|L|×tan(△ω/2). As the diffraction geometry shown in Fig. S2, $\omega_L > \omega_c > \omega_R$ means convex wafer bowing and compressive residual stress. On the other hand, the sample is concave bowing and under

tensile residual stress when $\omega_L<\omega_c<\omega_R$. The resolution of ω-scan was 0.004° in this work, corresponding to a resolution of wafer bowing of ~1.4 μm. After the extraction of wafer curvature κ, the residual stress could be calculated according to Stoney's Equation.

The wafer bowing measurement of sample grown on Sapphire substrate with thickness of 1 μm is shown in Fig. 1(c), and the measurement for the other two samples are shown in Fig. S3. It should be noticed that the diffraction intensity has not been normalized. The results of Fig. S3 indicate convex wafer bowing. The values of △ω are -0.132° and -0.072° for the 0.6-μm-thick and 1.2-μm-thick samples, corresponding to a residual stress of -0.91 GPa and -0.69 GPa, respectively.

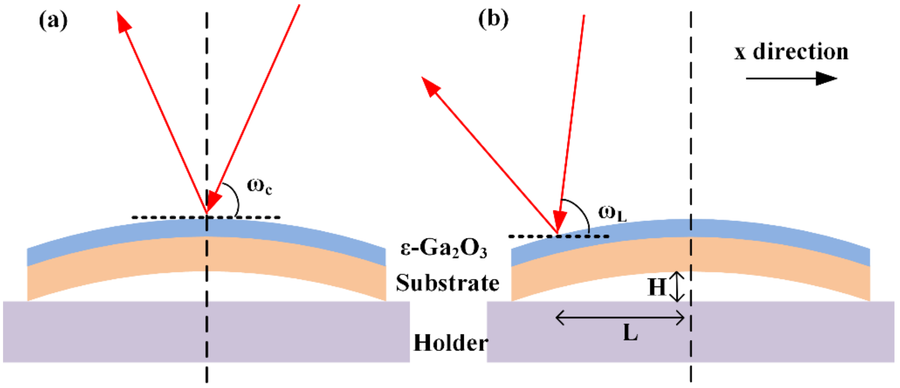

Figure S2. Diffraction geometry for ω-scan carried out at the (a) center and (b) left side of the wafer.

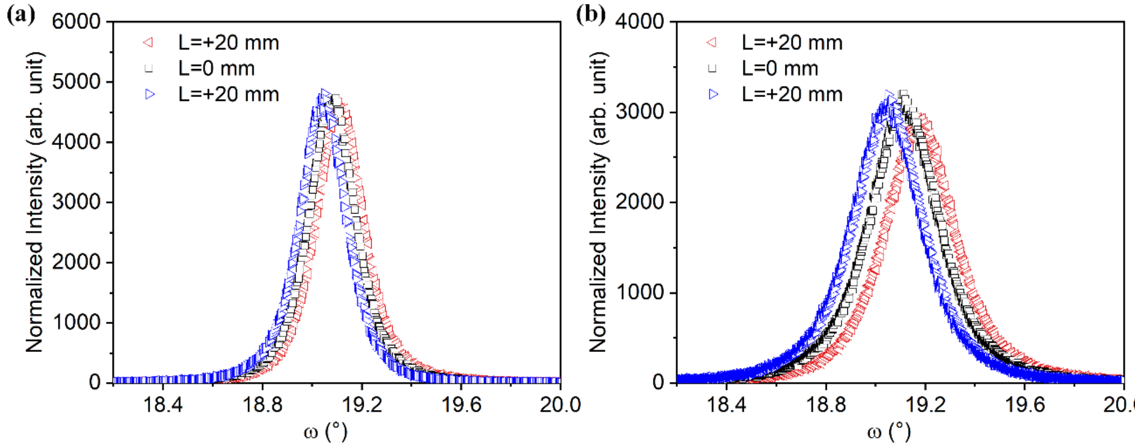

Figure S3. ω-scan of ε-Ga_2O_3(004) plane at different positions of the wafer for the (a) 0.6-μm-thick and (b) 1.2-μm-thick samples

3. SS-PFM measurement of ε-Ga_2O_3

The SS-PFM measurements were carried out to investigate the piezoelectricity of ε-Ga_2O_3. During the measurement, a DC voltage V_{dc} scanning between $-V_{max}$~$+V_{max}$ was applied on the sample. During the scanning, the V_{dc} was turned on and off, and an AC voltage V_{ac} was applied on the sample to excite piezoelectric oscillation of the sample when the V_{dc} was turned off [Fig. S4 (a)]. The amplitude of V_{ac} was set to be a constant of 0.8 V, while the maximum value of V_{dc} is changeable. Once the V_{ac} was applied on the sample, the sample would oscillate due to piezoelectricity and the phase and amplitude of oscillation was collected. As a comparison, SS-PFM measurement was also carried out on a GaN/AlN (2300/30 nm) heterostructure. The DC voltage scanned through -5 V~+5 V, -7 V~+7 V and -9 V~+9 V, and the results are shown in Fig. S4 (b). Compared to the ε-Ga_2O_3/AlN sample, the oscillation amplitude of the GaN/AlN structure was several times smaller and no hysteresis loop was observed, indicating a much smaller piezoelectric coefficient in GaN than that in ε-Ga_2O_3.

The SS-PFM measurements were carried out in four different positions on the transferred AlN/ε-Ga_2O_3 (50/360 nm) sample. The DC voltage scanned through -8 V → 0 V → +8 V → 0 V → -8 V for two times on each site. The detected amplitude and phase of piezoelectric oscillation is shown in Fig. S5.

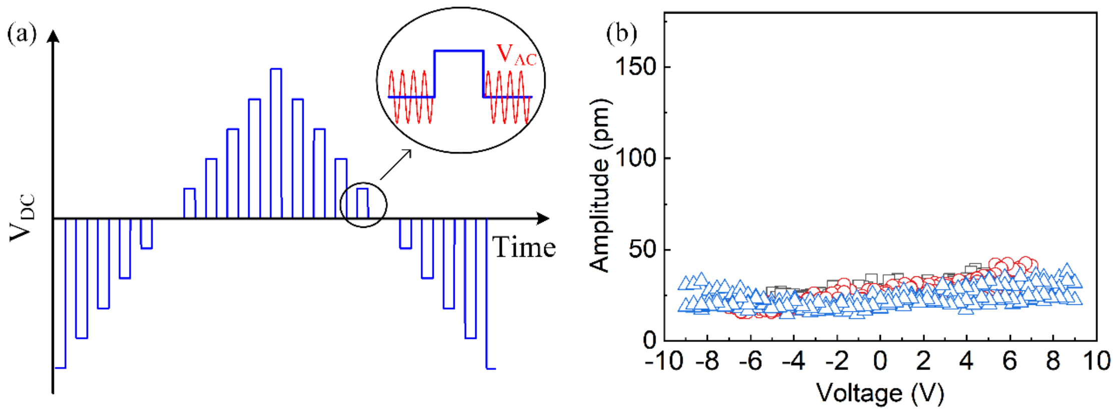

Figure S4. (a) Schematic diagram of the applied voltage during the SS-PFM measurement. (b) SS-PFM measurement of a transferred GaN/AlN (2300/30 nm) sample.

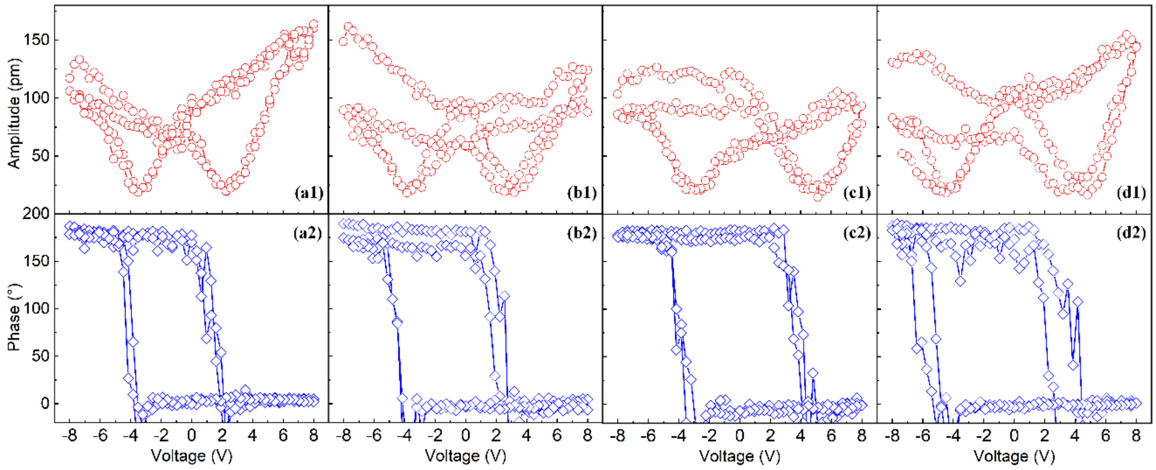

Figure S5. SS-PFM measurement of the ε-Ga_2O_3/AlN structure. (a)~(d) represent measurements in four different positions.

4. Structural properties of the AlScN thin film

The comercial AlScN sample used in this work was grown on Sapphire substrate with a Sc composition of ~40%. The thickness of the film is 1.2 μm. The XRD 2θ-scan in Figure S6(a) presents a diffraction peak at 35.87°, which belongs to the (002) plane of AlScN. The diffraction peak at 41.85° corresponds to Sapphire(006) plane. The HRXRD rocking curve of AlScN(002) plane in Figure S6(b) derives a FWHM of 0.04°, which means that the AlScN were also grown with high crystal quality.

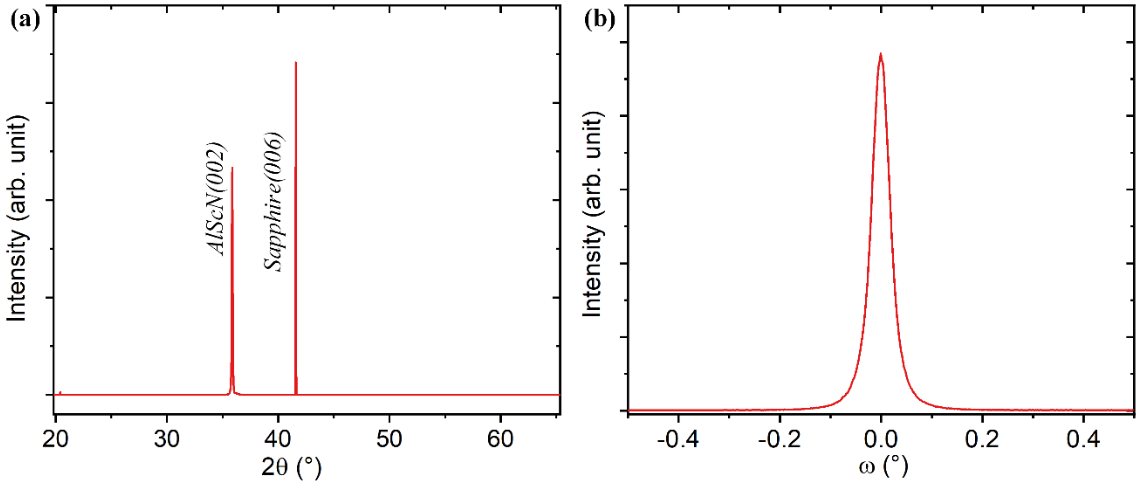

Figure S6. XRD (a) 2θ-scan and (b) rocking curve of AlScN thin film.

5. Extraction of mass density of ε-Ga_2O_3 by FEM calculation

The SAW resonators were constructed by IDTs with 80 pairs of periodic fingers, and the width W, spacing S and period λ of fingers are 0.6 μm, 0.6 μm and 2.4 μm, respectively. Since the length of the finger L_{IDT}=192 μm is much longer than the wavelength λ, it is acceptable to carried out FEM calculation in a simplified 2-dimension model [Fig. S7(a)]. Similarly, since the device was constructed by periodic fingers, only one period was considered in the FEM model. V_{AC}=1 V was applied on one electrode and the other electrode was grounded. Periodic boundary condition was applied on the left and right sides of the model. The thickness of sapphire substrate was set to be 10 μm. In fact, most of the energy are confined within the depth of λ under the surface. The bottom region (2 μm in thickness) of sapphire was set as a perfect matching layer (PML) which could absorb acoustic wave reaching the bottom of the model and avoid artificial reflection of acoustic wave. The calculated distributions of vibration are shown in Fig. S7(b)~(d).

There is no experimental report on the mass density of ε-Ga_2O_3. Since the density is a critical parameter determining the resonance frequencies of SAW, we have tried to determine the density of ε-Ga_2O_3 by comparing the measured and calculated resonance frequencies of the SAW resonator. The FEM calculation was carried out by using COMSOL software. The elastic constants C_{ij} of ε-Ga_2O_3 used for the calculation are shown in Table S1 (Phys. Rev. B 2016, 93, 115204). The piezoelectric stress constants e_{ij} of ε-Ga_2O_3 used for the calculation are shown in Table S2 (Mater. Res. Express 2018, 5, 036502). It should be noticed that the position of resonance frequency of IDT is affected by mass density, elastic constants and piezoelectric stress constants. However, the mass density and elastic constants play much more important roles since they determine the sound velocity directly. Piezoelectric stress constants have little influence on the frequency position.

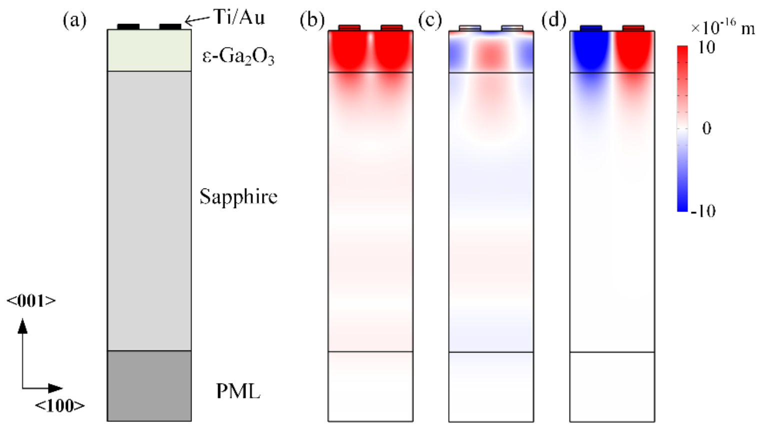

Figure S7. (a) Schematic diagram of the simplified 2-dimension model for FEM calculation. Distribution of (b) total displacement amplitude, (b) <100>-direction displacement and (b) <001>-direction displacement of Rayleigh mode vibration for the 1.2-μm-thick sample.

Generally, the velocity of acoustic wave is determined by the mechanical parameters with the form of $v_{acoustic} \propto (C_{ij}/\rho)^{1/2}$. Since the elastic constants are fixed as shown in Table S1, the density ρ becomes the only variable during the FEM calculation. The dependence of resonance frequencies on density for the 1.2-μm-thick sample is shown in Fig. S8(a). The acoustic wave velocities, as well as the resonance frequencies, decrease as the density increases. We define a deviation value to fit the calculated frequencies with the experimental values by:

$$Deviation = \left(\frac{1}{N}\sum_{i=1}^{N}\left(\frac{f_{i,exp} - f_{i,FEM}}{f_{i,exp}}\right)^2\right)^{1/2}$$

The subscript *i* represents different vibration modes. N is the number of observed modes and N=3 for the 1.2-μm-thick sample. The subscript *exp* and *FEM* means resonance frequencies derived from the measurement or FEM calculation. As is shown in Fig. S8(b), as the density increases, the deviation decreases at first and then increases, deriving a minimum value of 2.4% at ρ=4.8 gcm^{-3}.

Table S1 Elastic stiffness constants of ε-Ga_2O_3 (in GPa)

C_{11}	C_{12}	C_{13}	C_{22}	C_{23}	C_{33}	C_{44}	C_{55}	C_{66}
312.0	154.4	126.2	273.6	126.8	279.8	72.4	47.3	92.0

Table S2 Piezoelectric stress constants of ε-Ga_2O_3 (in Cm^{-2})

e_{31}	e_{32}	e_{33}	e_{24}	e_{15}
0.429	-0.198	0.844	0.196	0.712

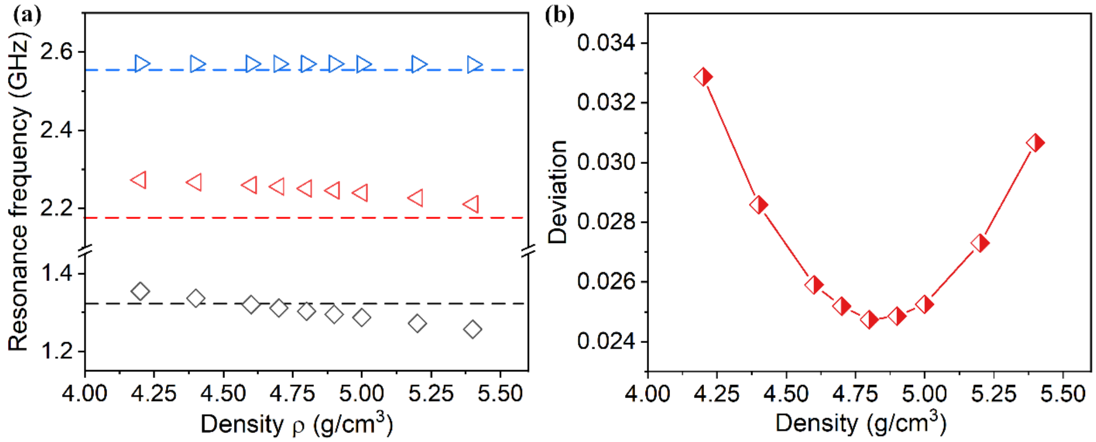

Figure S8. (a) Dependence of resonance frequencies on density for the 1.2-μm-thick sample. The measured resonance frequencies of different vibration modes at 1.32 GHz, 2.17 GHz and 2.54 GHz are indicated by dash lines in the figure. (b) Dependence of deviation on density.

6. Measurement of the bandgap of ε-Ga_2O_3

The bandgap of ε-Ga_2O_3 was extracted by optical method. The transmittance of the ε-Ga_2O_3 thin films with thickness of 0.6~1.2 μm are shown in Fig. S9(a). All the samples are transparent at the wavelength of 255~800 nm. The difference in transmittance reflects the surface roughness of different samples, which induces light scattering at the ε-Ga_2O_3/air interface and reduces the transmittance. The transmittance decreases abruptly below 255 nm due to the inter-band transition. The bandgap of ε-Ga_2O_3 E_g wa extracted by $(\alpha h\upsilon)^2=E_g-h\upsilon$, with $h\upsilon$ the energy of photon and α the absorption constant of light. The

extracted bandgap of ε-Ga_2O_3 is ~4.9eV [Fig. S9(b)].

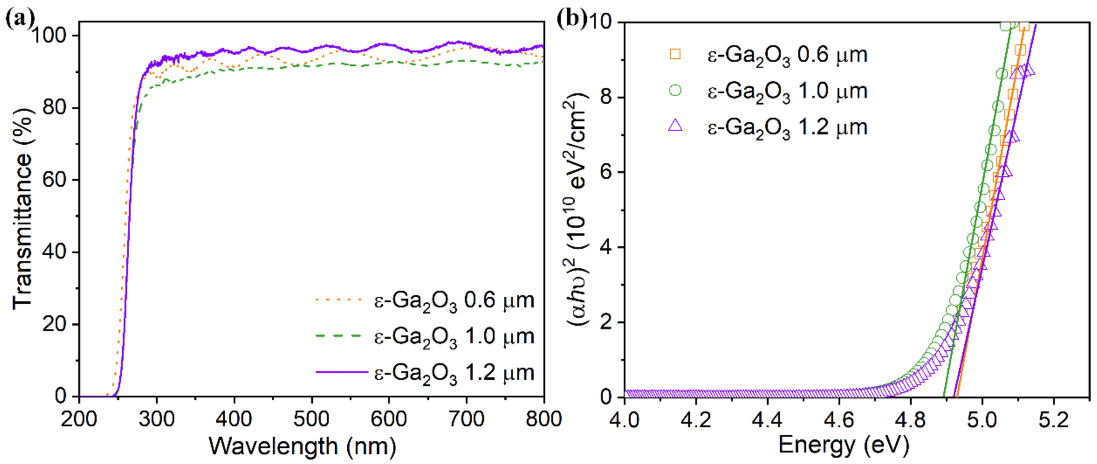

Figure S9. (a) Transmittance of ε-Ga_2O_3 thin films with different thickness. (b) Extraction of the bandgap of ε-Ga_2O_3 according to $(\alpha h\upsilon)^2 = E_g - h\upsilon$.

7. Calculation of modulus and sound velocities

The mechanical properties of orthorhombic ε-Ga_2O_3 was calculated by Voigt-Reuss-Hill model:

$$B_V=[C_{11}+C_{22}+C_{33}+2(C_{12}+C_{13}+C_{23})]/9$$

$$G_V=[C_{11}+C_{22}+C_{33}+3(C_{44}+C_{55}+C_{66})-(C_{12}+C_{13}+C_{23})]/15$$

$$D=C_{13}(C_{12}C_{23}-C_{13}C_{22})+C_{23}(C_{12}C_{13}-C_{23}C_{11})+C_{33}(C_{11}C_{22}-C_{12}C_{12})$$

$$B_R=D/[C_{11}(C_{22}+C_{33}-2C_{23})+C_{22}(C_{33}-2C_{13})-2C_{33}C_{12}+C_{12}(2C_{23}-C_{12})+C_{13}(2C_{12}-C_{13})+C_{23}(2C_{13}-C_{23})]$$

$$G_R=15/\{4[C_{11}(C_{22}+C_{33}+C_{23})+C_{22}(C_{33}+C_{13})+C_{33}C_{12}-C_{12}(C_{23}+C_{12})-C_{13}(C_{12}+C_{13})-C_{23}(C_{13}+C_{23})]/D+3(1/C_{44}+1/C_{55}+1/C_{66})\}$$

$$G=(G_V+G_R)/2$$

$$B=(B_V+B_R)/2$$

$$E=9BG/(3B+G)$$

$$v=(3B-2G)/2(3B+G)$$

with B, G, E and v the bulk modulus, shear modulus, Young's modulus and Poisson's ratio, respectively. The elastic stiffness constants C_{ij} are listed in Table S1. The calculated results are B=186.1 GPa, G=70.9 GPa, E=188.8 GPa and v=0.3309.

Neglecting the anisotropy of ε-Ga_2O_3, the longitudinal and shear sound velocities could be calculated by:

$$v_l = \sqrt{\frac{E(1-v)}{\rho(1+v)(1-2v)}}$$

$$v_s = \sqrt{\frac{E}{2\rho(1+v)}}$$

Since the density of ε-Ga_2O_3 has already been determined to be ρ=4.8 gcm^{-3}, the calculated results are v_l=7.6 kms^{-1} and v_s=3.8 kms^{-1}.
